# A new organ preservation solution for static cold storage of the liver. Amniotic fluid^1^

**DOI:** 10.1590/s0102-865020190040000002

**Published:** 2019-04-29

**Authors:** Başak Büyük, Tuba Demirci, Yasemen Adalı, Hüseyin Avni Eroğlu

**Affiliations:** I Assistant Professor, Department of Histology-Embryology, Faculty of Medicine, Çanakkale Onsekiz Mart University, Turkey. Conception and design of the study; acquisition, analysis and interpretation of data; histopathological examinations; manuscript writing.; II Assistant Professor, Department of Histology and Embryology, Faculty of Medicine, Ataturk University, Turkey. Scientific and intellectual content of the study.; III Assistant Professor, Department of Pathology, Faculty of Medicine, Çanakkale Onsekiz Mart University, Turkey. Histopathological examinations, statistics analysis.; IV Assistant Professor, Department of Physiology, Faculty of Medicine, Çanakkale Onsekiz Mart University, Turkey. Scientific and intellectual content of the study, manuscript preparation.

**Keywords:** Liver Transplantation, Amniotic Fluid, Pathology, Apoptosis, Rats

## Abstract

**Purpose:**

To evaluate the effect of amniotic fluid in liver preservation in organ transplantation, and compare it with standard preservation solutions.

**Methods:**

The groups consisted of Group 1: Ringer Lactate (RL) group, Group 2: HTK group, Group 3: UW group, Group 4: AF group. The livers of rats from Group 1, 2, 3, and 4 were perfused and placed into falcon tubes containing RL, HTK, UW, and AF solutions at +4‎°C, respectively. The tubes were stored for 12 hours in the refrigerator at +4°C. Tissue samples were taken at the 6^th^ and 12^th^ hours for histopathological examinations of the perfused livers, and storage solutions for biochemical analyzes at 6^th^ and 12^th^ hours.

**Results:**

AF was shown to maintain organ viability by reducing the number of cells undergoing apoptosis. Histopathological changes such as sinusoidal dilatation, hydropic degeneration, and focal necrosis were found to be similar to the groups in which the standard organ preservation solutions were used. Additionally, the results of INOS, IL-10, and TNF-α,which were evaluated immunohistochemically, have been shown to be similar to the UW and HTK groups.

**Conclusions:**

AF provided conservation similar to UW and HTK in the 12-hour liver SCS process. The fact that apoptosis values are comparable to standard preservation solutions supports the success of AF in the cold storage of the liver.

## Introduction

 Liver transplantation (LT) is a vital treatment option for patients with end-stage liver failure^1^ ^,^ ^2^. Despite new surgical techniques, intensive care units, and recent advances in immunosuppressive therapies, a significant problem for patients waiting for transplantation is organ shortage, and the deficit between pending patients and the number of organs obtained is increasing day by day^1^ ^,^ ^3^ ^-^ ^6^. Since the number of organs derived from donors is limited, many patients lose their lives while waiting for an organ^7^. Furthermore, postoperative transplantation failure still persists, and many patients cannot survive long enough for re-transplantation due to insufficient donors^2^. 

 In addition to the shortage experienced during organ supply, keeping the viability of the organs while transfer and transplantation are crucial^5^. Preservation of the liver received from the donor under ideal conditions up to the time of transplantation is the most critical step in determining whether the graft will be functional in the recipient^2^. This step is particularly crucial because the organs that will be transplanted in the distant centers are exposed to ischemia for a long time^2^.

 When the organ blood flow is cut off, metabolism must be slowed down in the cell that begins to deteriorate with ischemia. The main factor in slowing cell metabolism is hypothermia^8^. The process that starts at this stage is static cold storage (SCS). SCS is an organ preservation method used for all organs^5^. In cold storage, metabolic activity is reduced by 10 times and anaerobic metabolism and lactic acidosis increase; thus, the mitochondrial energy cycle stops^5^. Even with the most effective preservation solutions, SCS increases graft damage before transplantation^5^.

 Preservation solutions used in this phase help to preserve the organ without being damaged as its composition prevents the swelling of the cells^5^. Although many preservation solutions have been used in the last 20 years, the University of Wisconsin (UW) cold storage solution and histidine-tryptophan-ketoglutarate (HTK) solutions are the most commonly used ones^2^. Preservation solutions contain a wide variety of substances for the prevention of cellular swelling, stabilization of the cell membrane, induction of intracellular electrolyte balance, antioxidant, and cytoprotective effects^2^. The UW solution developed by Belzer has been an essential invention in this field^5^. UW has been accepted as the gold standard in liver transplantation since 1987^9^ ^,^ ^10^. It has been shown that the UW solution provides protection in the donor liver, 12-18 hours in clinical trials and 48 hours in experimental studies^3^. 

 The amniotic fluid (AF) is a protective liquid that fills the amniotic cavity which surrounds the fetus in intrauterine life and is required for fetal development and maturation^11^. Essentially, it protects the fetus from factors such as trauma and creates an appropriate environment for fetal growth and movement^11^. 98% of the AF content is made up of water and electrolytes; the rest is proteins, peptides, carbohydrates, lipids, amino acids, hormones, antimicrobial molecules, lactate, pyruvate, and growth factors^12^ ^,^ ^13^. The water in AF comes mainly from maternal plasma and reaches the amniotic cavity by passing the fetal membranes with osmotic and hydrostatic pressure^14^. Sodium and chloride, the primary electrolytes in the AF, are almost the same amounts as in the maternal serum. However, potassium, magnesium, calcium, and glucose are in lower concentrations than the mother serum^13^.

 Although most pregnant women have microorganisms in the AF, very few develop amniotic infections. The reason for this is that there are inhibitory substances that prevent the growth of microorganisms in the amniotic fluid. These substances include; lysozyme, beta lysine, transferrin, alpha defensin, peroxidase, bactericidal/permeability-increasing protein, calprotectin, a secretory leukocyte protease inhibitor, psoriasin, cathelicidin, and immunoglobulin^11^ ^,^ ^13^ ^,^ ^14^. 

 Studies have shown that as opposed to wounds in adults, fetal wounds are healed without leaving scar tissues. It was also confirmed that the rate of re-epithelisation was high in AF-treated injuries. It is claimed that the high amount of hyaluronic acid in AF may be responsible for this result^15^. 

 In another study, AF was found to increase mitosis and angiogenesis in AF-treated wounds. In this study, these effects were thought to occur due to growth factors such as fibroblast growth factor (FGF), epidermal growth factor (EGF), and transforming growth factor-beta (TGF-beta) existing in the amniotic fluid^16^. The amount of EGF increases in the 2^nd^ trimester in the human AF^13^. Again, TGF-alpha found in the AF plays an essential role in fetal bowel development. Also, TGF-beta is found in the AF and accelerates wound healing by stimulating cell migration. Immunoglobulin-A (Ig A) release, which is important for the immunity, also increases in the AF. Other vital substances in AF are insulin-like growth factor-1 (IGF-1), erythropoietin (EPO), and granulocyte colony-stimulating factor (G-CSF)^13^. 

 EPO is a hormone primarily secreted by the kidneys. It is responsible for regulating the number of circulating erythrocytes. In recent years, EPO receptors have been shown to be present also in the heart and brain in addition to the liver and kidneys. Thanks to these receptors, EPO acts as a cytokine, producing cytoprotective and anti-apoptotic effects, thereby preventing tissue damage^17^. EPO concentrations in the AF have similarities with the umbilical cord blood plasma concentration. Besides, the EPO concentrations in the AF increase further during hypoxia^18^. EPO, which increases 2-4 times in the mother during pregnancy and enters the AF, plays a protective role in acute and subacute tissue damage. The cytoprotective effect is due to its anti-inflammatory and anti-apoptotic properties^19^. Because of these protective effects, EPO has been used in some organ transplantation studies, directly^20^ or by adding into standard organ storage solutions^21^. 

 This study aimed to compare the use of AF in organ preservation and compare it with standard preservation solutions.

## Methods

 This study employed an experimental design with four randomization groups. The study was approved by the Çanakkale Onsekiz Mart University (ÇOMÜ) Experimental Animal Research Ethics Committee (Approval number: 2016-02-03). Animal procedures were performed according to the “Guide for the ‎Care and Use of Laboratory Animals” principles^23^. All steps of the study were conducted at the experimental research center of the university, open for supervision. 

 In this study, 40 male Wistar Albino rats weighing from 220-300 g were used. The rats were supplied by the Çanakkale Onsekiz Mart University Experimental Research Center. All rats were housed in pairs in appropriate cages in an animal room maintained at a standard humidity (45%-50%) and temperature (22±2°C) with 12 hours light and 12 hours darkness, and were fed with standard food and water ad libitum.

### Randomization

 The 40 male rats were randomly divided into four groups. Randomization was done by giving the rats sequential numbers and randomly assigning to groups using a random-numbers table.

### Acquisition of amniotic fluid

 AF was obtained from patients who were admitted to ÇOMÜ Obstetrics and Gynecology Clinic for term delivery, without any complications and who were not smoking, not using alcohol or any substance. AF was collected and centrifuged two times at 4000 rpm for 2 min without any time delay, and the sediment was removed each time. The supernatant obtained after centrifugation was filtered through pores of 0.22 microns.

### Experimental procedure

 The groups consisted of:

Group 1: Ringer Lactate (RL) group
Group 2: HTK group
Group 3: UW group
Group 4: AF group.

The surgical model used in the experiment was performed as previously described in the literature^2^ ^,^ ^22^. The rats were anesthetized with ketamine hydrochloride (50 mg/kg, Ketalar^®^, Pfizer, Turkey) and Xylazine (10 mg/kg, Rompun^®^, Bayer, Canada). Then a midline abdominal incision was made. After the portal vein was seen, it was entered with a cannula. Ringer Lactate (RL), HTK, and UW solutions and AF, which were previously stored at +4°C, were injected into the portal veins of rats in Group 1, 2, 3 and 4, respectively, and the liver was perfused. The hepatic vein was cut, and perfusion was continued until a clear liquid came from the vessel. Then, a hepatectomy was performed. After hepatectomy, rats were sacrificed with high dose anesthetics. The livers from Group 1, 2, 3, and 4 were placed into falcon tubes containing RL, HTK, UW, and AF solutions at +4‎°C, respectively. The tubes were stored for 12 hours in the refrigerator at +4 °C. Tissue samples were taken at the 6^th^ and 12^th^ hours for histopathological examinations of the perfused livers. Samples were also taken from storage fluids for biochemical analyzes at 6^th^ and 12^th^ hours. 

### Histopathological evaluation

 Tissue samples collected at the 6^th^ and 12^th^ hours after perfusion were put into 10% neutral buffered formalin without delay. At the end of the 48-hours fixation period, the tissues were subjected to routine tissue follow-up procedures. After the tissue processing protocol, livers were embedded in paraffin blocks, and sections at a thickness of ‎4-5 microns were obtained from the paraffin blocks with a microtome (Leica RM 2125 RTS) and stained with routine hematoxylin-eosin (H&E) method followed by assessments with a Zeiss AxioScope A1 brand light microscope. Histopathological assessments were completed and scored for centrilobular hydropic degeneration, sinusoidal dilatation, and focal necrosis, which employ a scale ranging from 0 to 3 as follows:

 0= No changes found; 1= Mild change; 2= Moderate change; and 3= Severe change.

### Immunohistochemical evaluation

 Immunohistochemical (IHC) methods with TNF-α (Tumor necrosis factor alpha), IL-10 (Interleukin 10), and iNOS (Inducible nitric oxide synthetase) primary antibodies were used to show injury in the liver. After fixation in 10% neutral buffered formalin and routine histologic monitoring, the liver tissues embedded in paraffin blocks were sliced into 4-micron sections with a microtome and placed on adhesive slides. Antigen retrieval method was applied to the sections, which were left at 65°C for 1 hour and deparaffinized in xylene before passing through an alcohol series for rehydration. Later, the sections were left in 10 mM EDTA (Thermo Scientific LOT: Ax201208) for 20 minutes in a 200-watt microwave oven and then cooled at room temperature for 20 minutes. After cooling, each slide had sections outlined with a PAP pen. Then, 3% H_2_O_2_ (Thermo Scientific LOT: HP31685) was dropped onto the specimens and left on the place for 15 minutes. Later, the samples were washed in phosphate buffered saline (PBS) with a pH of 7.4. 

 Lastly, all sections were incubated with anti-TNF-α (EMD Millipore Corporation, clone 13F9.1, Lot #Q2573230), anti-IL-10(Santa Cruz Biotechnology, INC, sc-28343, Lot#I1316), and anti-iNOS (Cell Signaling Technology, Lot#12242) primary antibodies, marked with DAB chromogen (Thermo Scientific LOT: HA33805) and counterstained with Harris hematoxylin before being covered.

 INOS staining in hepatocytes was evaluated as follows^2^:

Grade 0: Less than 5% cell staining;
Grade I: 5% -25% cell staining;
Grade II: 25% -50% cell staining;
Grade III: more than 50% cell staining.

### Apoptosis assessment with TUNEL (Terminal deoxynucleotidyl transferase dUTP nick end labeling) method

 The terminal deoxynucleotidyl transferase dUTP nick end labeling (TUNEL) staining was used to detect apoptosis of the liver tissue. Four µm thick sections were cut from each paraffin block. After dewaxing, hydration, and serum blocking, the ApopTag Peroxidase in situ Apoptosis Detection Kit (S7100, Merck Millipore, Darmstadt, Germany) was used according to the manufacturer’s protocol. In order to determine the apoptotic index (AI), five randomly selected regions of each section were chosen under x400 magnification. Cells stained brown or black were judged as TUNEL-positive apoptotic cells. The AI of hepatocytes was determined as the percentage of TUNEL positive cells with respect to the total number of cells counted using the formula:

Apoptoticindex(AI)=(Numberofpositivecells/Totalnumberofcellscounted)x100.

### Blinding

 In this study, blinding was applied at the stage of the histopathological investigation. Histopathological assessments were performed by two histopathologist who had no ‎knowledge about the groups. ‎

### Biochemical evaluation

 Two ml samples were taken from the preservative fluid where the subjects of each group had their own liver tissue, at the 6^th^ and 12^th^ hours, to the biochemistry tubes without anticoagulant. The levels of aspartate aminotransferase (AST) and alanine aminotransferase (ALT) were measured in the biochemical laboratory using enzymatic colorimetric methods. 

### Statistical analysis

 Data were analyzed using the SPSS Package Program version 15.0. Mean and standard deviations were used in the presentation of descriptive data. The Shapiro-Wilk test was used to determine the normal distribution of the variables. The Chi-Square test was used for comparing hydropic degeneration, sinusoidal dilatation, and focal necrosis degrees among the groups. One way ANOVA and the independent samples t-test were used to compare TUNEL score and ALT and AST values between the groups. The paired t-test was used for the assessment of the variables at the 6^th^ and 12^th^ hours. A p-value below 0.05 was considered statistically significant.

## Results

 The mean and standard deviations of the numerical variables are given in Table 1. 

**Table 1:** Mean and standard deviations (SD) of the parametric data compared across groups.

	Group 1 Mean±SD	Group 2 Mean±SD	Group 3 Mean±SD	Group 4 Mean±SD
TUNEL 6^th^ hour	21.9±4.4	11.2±4.4	10.0±3.5	11.2±4.0
TNF alpha 6^th^ hour	1.8±0.6	1.7±0.7	1.8±0.6	1.7±0.7
IL-10 6^th^ hour	0.50±0.7	1.0±0.7	1.0±0.7	0.9±0.7
iNOS 6^th^ hour	1.5±0.5	1.0±0.7	0.9±0.6	0.9±0.6
ALT 6^th^	190.8±193.0	32.2±47.8	160.5±83.9	93.2±141.5
AST 6^th^	371.5±162.1	214.4±158.7	366.5±219.6	227.9±153.0
TUNEL 12^th^	25.0±5.6	14.2±5.0	11.9±3.6	142.0±3.8
TNF alpha 12^th^ hour	1.8±0.6	1.7±0.8	1.4±0.5	1.7±0.7
IL-10 12^th^ hour	0.90±0.74	2.20±10.3	2.10±0.74	2.10±0.74
iNOS 12^th^ hour	2.5±1.0	2.0±0.7	1.8±0.8	2.0±0.7
ALT 12^th^ hour	331.5±128.6	76.4±64.2	477.66±464.4	259.7±261.9
AST 12^th^ hour	992.8±543.1	540.3±528.1	658.8±311.0	714.3±417.8

### Histopathological evaluation

 Histopathological grades of all groups were placed in Table 2. 

**Table 2:** Grades of the histopathologic and immunohistochemical parameters belong to all groups.

Groups	Grades	Immunohistochemistry						Histopathology					
		iNOS		TNF-α		IL-10		Hydropic degeneration		Sinusoidal Dilatation		Focal Necrosis	
		n		n		n		n		n		n	
		6^th^ hour	12^th^ hour	6^th^ hour	12^th^ hour	6^th^ hour	12^th^ hour	6^th^ hour	12^th^ hour	6^th^ hour	12^th^ hour	6^th^ hour	12^th^ hour
Group 1 (n=10)	0	0	1	0	0	0	0	1	1	4	1	5	4
	1	5	0	3	1	4	3	2	2	4	4	2	2
	2	5	2	6	6	3	2	5	3	0	2	0	2
	3	0	7	1	1	3	5	2	4	2	3	3	2
Group 2 (n=10)	0	2	0	0	0	2	0	4	2	5	4	0	7
	1	6	2	4	5	6	5	4	4	4	5	8	2
	2	2	6	5	3	2	4	1	3	1	0	1	0
	3	0	2	1	2	0	1	1	1	0	1	1	1
Group 3 (n=10)	0	2	0	0	0	3	0	3	3	4	3	6	4
	1	7	4	3	6	5	5	4	5	5	4	2	4
	2	1	4	6	4	2	4	2	0	0	2	2	0
	3	0	2	1	0	0	1	1	2	1	1	0	2
Group 4 (n=10)	0	2	0	0	0	3	0	4	2	4	7	6	4
	1	7	2	4	4	6	5	4	6	5	1	2	4
	2	1	6	5	5	1	4	1	0	1	1	2	1
	3	0	2	1	1	0	1	1	2	0	1	0	1

 When the data for the sinusoidal dilatation at the 6^th^ hour were considered, there was no statistical significance between the groups (p>0.05). Yet, when the 12^th^-hour data were analyzed, a statistically significant difference was observed between the groups (p=0.033). Accordingly, sinusoidal dilatation in Group 1 was significantly more than that of the Group 4. There was no statistically significant difference between the groups regarding the 6^th^ and 12^th^ hour sinusoidal dilatation (p> 0.05). 

 There was no statistical difference between the groups in the evaluation of the 6th and 12th hours of centrilobular hydropic degeneration and focal necrosis (p> 0.05). Similarly, there was no statistical significance in both histopathological parameters at the 6th and 12th hours of the study (p> 0.05). 

### Apoptosis evaluation with TUNEL staining

**Figure 1:**
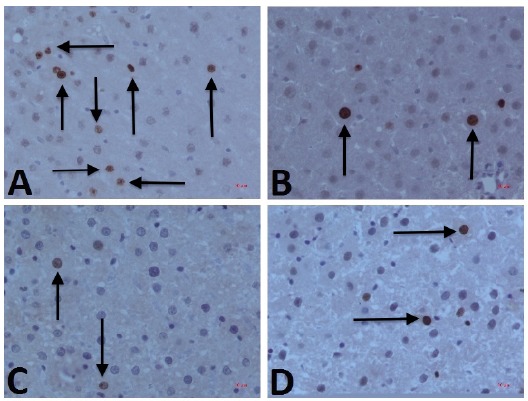
TUNEL Staining images of Group 1, 2, 3 and 4 were **A**, **B**, **C** and **D**, respectively. Apoptotic cells were shown with arrows. (Magnification x400)

 TUNEL staining images of all groups were placed in Figure 1. When t-test was used for the independent variables of Group 1, 2, 3, and 4, AI values examined at the 6^th^ and 12^th^ hours were significantly different in Group 1 (p values both at 6^th^ and 12^th^ hours were <0.001). 

 There was no statistically significant difference between the AI values of Group 2 and Group 3 and 4 at the 6^th^ and 12^th^ hours (p values 0.511, 1.0, 0.254, and 1.0, respectively). When the group 3 and 4 were compared with the t-test, there was no statistically significant difference between the values of AI (p values 0.484 and 0.180, respectively).

 Compared between 6^th^ and 12^th^ hours, the AI levels were significantly higher in the Group 4 only (p = 0.025). 

### Immunohistochemical evaluation

 Immunohistochemical grades of all groups were placed in Table 2. When the iNOS immunohistochemistry was evaluated, it was found that the values at both the 6^th^ and the 12^th^ hours in Group 1 were significantly higher compared to Groups 2, 3, and 4. However, in the statistical analysis, only the 6^th^-hour data showed a significant difference between Group 1 and Group 3, in favor of the first group (p = 0.013).

 When the 6^th^ and 12^th^ hour iNOS values of Group 1 were compared, it was found that the 12^th^-hour value was statistically higher (p=0.032). No statistically significant relation was found in the comparison of the other groups at the 6^th^ and 12^th^ hours (p>0.05). Similarly, there was no significant difference between the groups at the 6^th^ and 12^th^ hours of TNF-α and between the 6^th^ and 12^th^ hours within all groups (p> 0.05). 

**Figure 2:**
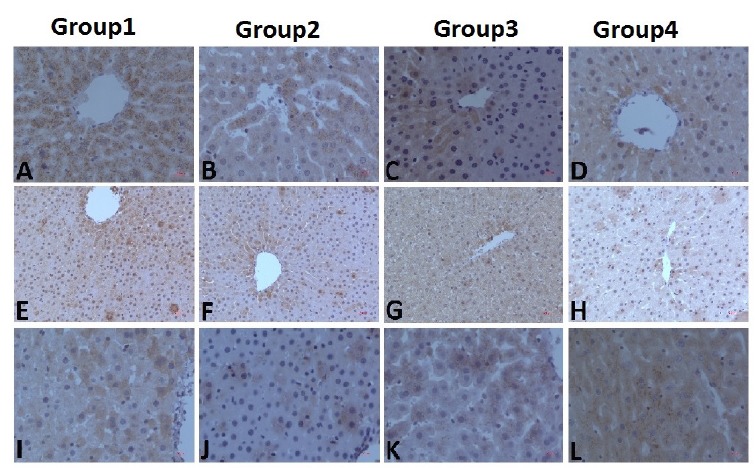
IHC stainings of all groups were shown. While **A**, **B**, **C** and **D** are iNOS staining images, **E**, **F**, **G** and H are TNF-α and **I**, J, **K** and **L** are IL-10 staining images of groups. Magnification for A, B, C, D, I, J, K and L is x400, for E, F, G and H is x200.

 When the 6^th^-hour data for IL-10 was evaluated, although the mean of the Group 1 was somewhat lower than Group 2, 3, and 4, this difference was not statistically significant (p values 0.121, 0.121, and 0.232, respectively). When the 12^th^-hour data for IL-10 were examined, Group 1 had significantly lower levels than the other groups (p values for Group 2, 3 and 4 were 0.005, 0.002, and 0.002, respectively). No statistically significant difference was found between the IL-10 values of Group 2 compared with Group 3 and 4 (p = 0.806 for both comparisons) and also between the IL-10 values of Group 3 and Group 4 (p = 1.0). In the comparison of the data at the 6^th^ and 12^th^ hours, the 12^th^-hour data were statistically higher than the 6^th^-hour data in all groups (p values were 0.005, <0.001, <0.001, and <0.001, respectively). Figure 2 has shown the IHC images of all groups.

### Biochemical evaluation

 When the 6^th^-hour data were evaluated for ALT, (one of the parameters we frequently use in routine practice to assess liver damage) Group 3 had significantly higher values compared to Group 1 and Group 2 (p values 0.030 and 0.001, respectively). Also, the 12^th^-hour values were significantly higher in Group 3 compared to Group 1 and 2 (p values <0.001 and 0.023, respectively). There was no statistically significant difference between the groups at the 6^th^ and 12^th^-hours (p> 0.05). AST values at the 6^th^-hour were significantly different only between Group 1 and 2 (p=0.042).

 When the mean AST were compared between the 6^th^ and 12^th^ hours within groups, 12^th^-hour values were significantly higher in Group 1, 2, and 3 (p values 0.010, 0.025, and <0.001, respectively). However, the difference was not significant for group 4 (p> 0.05).

## Discussion

 At the end of this study, the AF was shown to protect the liver throughout the 12-hour cold ischemia period in rats, comparable to standard organ preservation solutions, and maintained organ viability by reducing the number of cells undergoing apoptosis. Histopathological changes such as sinusoidal dilatation, hydropic degeneration, and focal necrosis were found to be similar to the groups in which the standard organ preservation solutions were used. Additionally, the results of INOS, IL-10, and TNF-alpha, which were evaluated immunohistochemically, have been shown to be similar to the UW and HTK groups.

 The AF perfectly protects and stores the fetus during pregnancy and serves as a barrier against infections^11^ ^-^ ^14^; it supports the development and maturation of fetal organs throughout the intrauterine life. Studies have shown that the addition of EPO to preservation solutions during the transplantation process protects the organ from ischemia-reperfusion injury^20^ ^,^ ^21^. In our study, the number of apoptotic cells decreased in the AF-perfused liver tissue compared to the control group, an effect similar to using RL, UW, and HTK. Additionally, the level of sinusoidal dilatation was higher in the control group than in the AF group. This effect may be due to the protective properties of EPO in the AF on the liver. Besides, since the electrolytes contained are similar to the plasma levels, AF is seen as a balanced solution. Therefore, AF may have been effective in protecting and preventing damage to the cells during the SCS process.

 Apoptosis is the most important parameter affecting organ viability and post-transplant success. Preserving the viability of hepatocytes enables the patient to live a healthier life after reperfusion. Standard preservation solutions have been developed to reduce hepatocyte damage and store the liver for a longer time. It was shown that UW and HTK significantly reduce the number of apoptotic cells in the liver during the SCS process compared to the control group^2^. In our study, apoptosis values were significantly decreased in groups using HTK, UW, and AF compared to the control group using RL. The AI values of the AF group were similar to the UW and HTK perfusion groups. This result suggests that AF may protect the liver similarly to standard solutions during cold ischemia and increase viability. We found that sinusoidal dilatation evaluated at the 12^th^ hour was significantly reduced in the AF group compared to the control group, which supports the success of AF in liver preservation.

 Nitric oxide (NO) is a potent vasodilator in the body, produced by two different nitric oxide synthases (NOS): the constitutive NOS (cNOS) and inducible NOS (iNOS). cNOS is mainly expressed in the brain (nNOS) and endothelium (eNOS), whereas iNOS is expressed in many cells in the liver, such as Kupffer cells, hepatocytes, and vascular smooth muscle cells^24^. It is known that iNOS secretion is increased during liver damage, and thus, NO produced in excess reacts with superoxide anions and forms peroxynitrite. Peroxynitrite, in turn, increases lipid damage, apoptosis, and necrosis and increases liver damage^25^. In a previous study, comparing immunohistochemical iNOS values in the liver SCS model using RL, UW, and HTK perfusion, iNOS was shown to be higher in the RL perfusion group compared to the other groups^2^. In the present study, the values of immunohistochemical iNOS were higher in the control group at the 6^th^ and 12^th^ hours compared to the other groups. The results of immunohistochemical iNOS evaluation were significantly lower in Group 4 at 6 hours, especially in patients with AF perfusion. Additionally, the results of iNOS at the 6^th^ and 12^th^ hours in Group 2 and 3 were similar to those of the AF group. This showed that AF could protect the liver from cold ischemic injury and the use of perfusion solution in transplantation surgery would have similar results with standard solutions such as UW and HTK. In the group with AF perfusion, the iNOS values were found to be significantly lower, suggesting that the amount of NO in the medium remained within the normal range, preventing the increase of lipid peroxidation and apoptosis. In our study, the 12^th^ hour iNOS values were significantly higher in the control group who underwent RL perfusion compared to the 6^th^ hour. This increase in iNOS may increase the number of cells leading to apoptosis, which may decrease organ viability and lead to a decreased post-transplantation success.

 TNF-α is a proinflammatory cytokine synthesized from hepatocytes and Kupffer cells in the liver^26^. In the current study, the immunohistochemical TNF-α values of Group 2, 3, and 4 at the 6^th^ and 12^th^ hours were lower than the control group, but these results were not statistically significant. This may be because reperfusion was not performed in the liver during the 12 hours SCS. The release of inflammatory cytokines can increase by reperfusion, which has been evaluated in previous studies^26^ ^,^ ^27^. On the other hand, IL-10 is known as an anti-inflammatory cytokine. In many organs, IL-10 has been shown to reduce ischemia-reperfusion (I/R) damage and have cytoprotective effects^26^. The increase of IL-10 in the tissue causes a decrease in many proinflammatory cytokines including TNF-α. In this way, IL-10 can reduce hepatocyte damage in the liver^27^. In this study, IL-10 levels evaluated immunohistochemically were significantly higher in the UW, HTK, and AF groups compared to the control group using RL. This indicates that hepatocyte injury is reduced in these groups due to the cytoprotective effect of IL-10. In this way, the liver tissues can be protected more effectively during the SCS process. Also, the AI values were lower in these groups compared to the control group. The fact that IL-10 values are close to UW and HTK groups in the AF group and there is no statistical difference between these groups shows that in organ preservation, AF can produce similar results as standard solutions can do.

 ALT and AST are the best known and widely used biochemical markers in the evaluation of hepatic damage^28^ ^,^ ^29^. In this study, the levels of ALT and AST measured at the 6^th^ and 12^th^ hours of organ storage in the cold ischemia process were examined. There was no significant increase in the ALT levels from 6 to 12 hours (p> 0.05). However, AST was significantly higher at the 12^th^ hour in Group 1, 2, and 3 (p values 0.010, 0.025, and <0.001, respectively). These results indicate that AF is even more successful than standard solutions for the long-term SCS of the liver.

## Limitations

 Successful storage of the liver in the SCS process results in a reduction in reperfusion injury. In this study, we did not reperfuse the liver in the cold ischemia model. Therefore, I/R damage assessment and post-transplant success could not be detected. Some intracellular mechanisms in the transplantation process contribute to cell damage. ATP depletion during ischemia causes loss of the transcellular electrolyte gradient, activation of free calcium influx and subsequent activation of phospholipases. Cell swelling and lysis occur as a result of all these events^5^. After ischemia-reperfusion, toxic molecules are produced primarily in the form of reactive oxygen species (ROS), leading to I/R damage^5^ ^,^ ^30^. In our study, besides the lack of, we also did not study the oxidative stress markers. Hence, we are unable to assess the toxic effects of ROS, which is another limitation of our study. 

## Conclusions

 Amniotic fluid provided conservation similar to UW and HTK in the 12-hour liver SCS process. The fact that apoptosis values are comparable to standard preservation solutions supports the success of AF in the cold storage of the liver.
